# Academic performance, self-reported motivation, and affect in higher education: the role of basic psychological need satisfaction

**DOI:** 10.3389/fpsyg.2025.1519454

**Published:** 2025-02-14

**Authors:** Mauricio González-Arias, Paula Dibona, Benjamín Soto-Flores, Andrés Rojas-Puelles, Massimo Amato, Diego Álvarez-Trigo, Rodrigo Castillo

**Affiliations:** ^1^Departamento de Psicología, Universidad de La Serena, La Serena, Chile; ^2^Departamento de Artes y Letras, Universidad de La Serena, La Serena, Chile; ^3^Departamento de Educación, Universidad de La Serena, La Serena, Chile

**Keywords:** self-determination theory, basic psychological needs, affect, higher education, students, motivation, academic performance

## Abstract

**Introduction:**

Higher education is a milestone in students’ lives; however, it often comes with various challenges. In this context, Basic Psychological Needs Theory emerges as a framework to understand a series of significant factors that influence students’ academic experiences, such as motivation and affect. Although there are studies that assess the association between basic psychological needs (BPN), motivation, affect and academic performance separately, there is a lack of research integrating all these variables in a higher education context. The first objective of the study was to evaluate the differences in BPN satisfaction, positive and negative affect, and academic performance between the courses perceived as the most motivating and those perceived as the least motivating. The second objective of the study was to examine the relationship between the studied variables.

**Methods:**

This non-experimental cross-sectional study included a sample of 148 higher education students from Chile. Paired sample t-tests were performed to compare the levels of the study variables between the courses, followed by structural equation modeling (SEM).

**Results:**

Findings for the t-tests reveal that courses considered the most motivating showed higher positive affect, lower negative affect, higher BPN satisfaction, and better academic performance. Results obtained through the SEM show that BPN satisfaction has an indirect effect on academic performance, mediated by affect and self-reported motivation. In addition, a direct effect from negative affect to academic performance was found.

**Discussion:**

These results contribute to a better understanding of how BPN satisfaction influences the academic performance of university students, and reinforce the usefulness of Self-determination Theory (SDT) in explaining motivational and affective phenomena in higher education.

## 1 Introduction

Higher education represents a significant milestone in students’ lives, marking the beginning of a phase of academic and personal growth (Leow et al., 2023). However, this period also brings challenges for higher education institutions, with student motivation being one of the most prominent (Brahm et al., 2017). Creating a motivating class is of paramount importance because, as recent studies highlight, motivation has a significant effect on students’ engagement (Karimi and Sotoodeh, 2019), self-efficacy (Li et al., 2023), and well-being (Tang et al., 2021).

In this context, Self-Determination Theory (SDT) provides a robust theoretical framework for understanding how the satisfaction of basic psychological needs (BPN)—autonomy, competence, and relatedness—impacts variables such as motivation and academic performance, which are fundamental to academic success (Ryan and Deci, 2001, 2020). In recent years, SDT has been widely studied within the educational context (e.g., Carmona-Halty et al., 2019; Oram and Rogers, 2022; Fierro-Suero et al., 2022), highlighting its significance in understanding academic learning and life satisfaction (Shi et al., 2024). Despite advancements in this field, a significant gap remains in understanding how these mechanisms function among university students in Latin America, particularly in Chile.

Basic Psychological Needs Theory, a sub-theory of SDT, posits the existence of three universal needs—autonomy, competence and relatedness—that are necessary for optimal psychological development (Ryan and Deci, 2017). As Ryan and Deci (2017) propose, autonomy refers to the need to independently regulate one’s actions and experiences, competence relates to the necessity of feeling proficient and effective, and relatedness entails the need for social connection. Satisfying these needs fosters proactive, prosocial and growth-oriented inclinations, promotes healthy adjustment, and contributes to overall well-being (Vansteenkiste et al., 2023).

The relevance of BPN satisfaction for positive educational outcomes is well established. Fulfillment of these needs positively predicts positive affect, negatively predicts negative affect, and enhances motivation, which in turn improves academic engagement and performance (Schutte and Malouff, 2021; Méndez-Aguado et al., 2020; Aydın and Michou, 2020; Liu et al., 2024). In an educational setting, motivation is particularly relevant, as SDT posits that enhancing it can lead to increased student achievement (Ryan and Deci, 2020).

Consistent with Basic Psychological Needs Theory, the association between BPN and motivation has been previously shown (e.g., Del Valle et al., 2025; Basileo et al., 2024; Lombas and Esteban, 2018). This latter construct, defined as the energy that drives people to choose, prioritize and act on their desires (Dörnyei and Ottó, 1998), is a crucial cognitive component for effective learning activities (Hurtado-Bermúdez and Romero-Abrio, 2023).

According to SDT (Ryan and Deci, 2020), motivation is classified into different types along a spectrum based on their degree of self-determination. At the most self-determined end lies intrinsic motivation, defined as the inherent drive to seek new learning experiences and apply previously acquired knowledge. Moving along the spectrum, extrinsic motivation reflects actions driven by the integration of external regulations with personal values and needs. At the least self-determined end is amotivation, characterized by a lack of intention or willingness to act.

Another theory that explores different aspects of motivation is the Achievement Goal Theory (Elliot and Hulleman, 2017). This theoretical framework states that different types of goals lead to different patterns of affect, cognition and behaviors, and thus, different academic outcomes. The mastery goals emphasize developing skills and achieving mastery of a task, while the performance goals center on showcasing competence in comparison to others. In this context, research has shown that mastery goals are associated with an adaptive motivational pattern, marked by a tendency to seek challenges and demonstrate strong persistence when facing obstacles, while performance goals are linked to avoiding challenges and displaying low persistence (Dweck, 1986).

Recent research has demonstrated that students’ motivation within the classroom is significantly influenced by their emotions (Vaculíková, 2021; Florescu et al., 2023; Wang et al., 2024), underscoring the increasing importance of the latter variable in educational contexts (Schutz and Pekrun, 2007). Emotions, as natural reactions that impact an individual’s mental state, not only condition responses to various situations (Méndez-Aguado et al., 2020), but also play a pivotal role in everyday life.

Emotions are a fundamental type of affective state that arise as reactions toward specific goals, or perceiving changes in relation to those goals (Batson et al., 1992). In contrast, affect is a broader construct, defined as a relatively stable inclination to experience specific moods and emotions across various contexts (Grzybowski et al., 2021). Watson et al. (1988) addressed affect through a two-dimensional model, distinguishing between Positive Affect and Negative Affect. Positive Affect is associated with pleasant subjective states, whereas Negative Affect encompasses a broad spectrum of distress, such as guilt, fear, or irritability (Schutz and Pekrun, 2007; Shiota et al., 2021; Watson et al., 1988). This widely validated model (Díaz-García et al., 2020) considers both dimensions as fundamental components of psychological well-being, as both negative affect and positive affect mediate the relationship between psychological need satisfaction and outcomes such as mental health and academic performance (Schutte and Malouff, 2021).

Crucial to understanding affect are their key components. Two pivotal dimensions in the conceptualization of affect are valence and arousal (Russell, 1980, 2003). Valence refers to the level of pleasantness associated with an event, which can be positioned along a continuum ranging from negative to positive; while arousal or intensity is defined as the level of autonomic activation, ranging from low to high (Bestelmeyer et al., 2017). In this sense, it is believed that positive activating emotions, such as enjoyment, increase motivation, while negative deactivating emotions, such as boredom, decrease it (Pekrun et al., 2007).

Affect has been shown to indirectly influence academic performance (Rodríguez-Muñoz et al., 2021), while motivation has a direct impact on this variable (Méndez-Aguado et al., 2020). Academic performance, usually evaluated through grades, is the benchmark by which student competence is measured (Reed, 2009). Poor academic performance not only has psychological consequences for students but also social and familial repercussions (Najimi et al., 2013).

Despite existing research, comprehensive studies integrating basic psychological needs, affect, motivation, and academic performance in higher education are limited. While previous studies have explored these variables individually or in pairs (e.g., Rodríguez-Muñoz et al., 2021; Schutte and Malouff, 2021), few have examined their interactions within a cohesive structural model. This study aims to address this limitation.

The first objective of this investigation was to evaluate the differences in BPN satisfaction, positive and negative affect, and academic performance between the courses perceived as the most motivating and those perceived as the least motivating by university students. Consistent with SDT and previous research, it was hypothesized that the most motivating courses would exhibit higher levels of BPN satisfaction, academic performance, and positive affect, as well as lower levels of negative affect.

**Figure 1:**
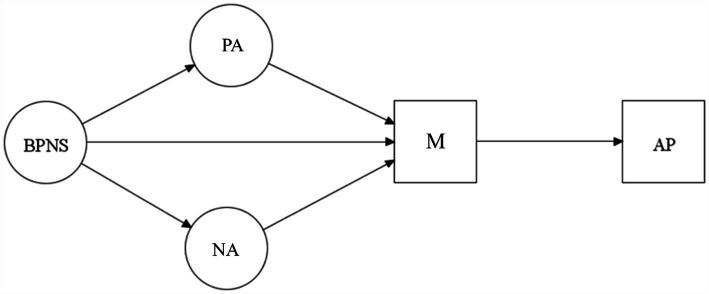
General model representing the second hypothesis of the study. BPNS, basic psychological need satisfaction; PA, positive affect; NA, negative affect; M, self-reported motivation; AP, academic performance. This Figure was created using Mplus 8.11 (Muthen and Muthen, 2024).

The second objective of the study was to explore the relationships among the study variables using a structural model. As depicted in Figure 1, it was hypothesized that BPN satisfaction positively predicts motivation and positive affect, while negatively predicting negative affect. Positive affect was expected to positively predict motivation, whereas negative affect was anticipated to negatively predict to it. Finally, motivation was expected to positively predict academic performance. This model is grounded in evidence supporting the predictive roles of affect and motivation in educational outcomes (Rodríguez-Muñoz et al., 2021; Méndez-Aguado et al., 2020).

Understanding the pathways linking motivation, affect, and performance is essential for designing effective educational interventions. Examining these dynamics in the context of higher education in Chile offers valuable insights into how cultural and contextual factors influence these relationships. Addressing this gap is crucial for developing culturally tailored strategies that enhance student well-being and academic success in Latin America. Additionally, the findings will contribute to theoretical and empirical advancements by shedding light on how psychological needs, emotions, and motivation interact to shape academic outcomes. This integrated approach provides a robust framework for improving educational practices and outcomes in higher education.

## 2 Methods

### 2.1 Subjects

The inclusion criteria for participation in the present study were: (1) being a higher education student; (2) being enrolled in a program of humanities, in line with the structure of academic programs in Chile (Pedagogy, Psychology, Music, Arts, Literature, or Law); and (3) being in either the first or fourth year of study. The careers considered were limited to the aforementioned in order to ensure greater homogeneity within the sample, allowing for more meaningful comparisons across participants from different academic backgrounds. Similarly, the sample was intentionally delimited to first- and fourth-year students to minimize potential biases between new and senior students during data collection, and to ensure comparability across academic levels. A convenience sampling method was employed to facilitate participant recruitment, leveraging the goodwill of instructors who granted access to students in their courses.

This study included a nonrandom sample of 148 higher education students from various disciplines: pedagogy (35%), psychology (33%), law (29%). The majority of participants were from the Coquimbo region (94%), with 6% from other regions of Chile. Among the participants, 84 (56.8%) were first-year students and 64 (43.2%) were fourth-year students. In terms of gender distribution, 56 (37.8%) identified as male, 87 (58.8%) as female, and 5 (3.4%) did not disclose their gender. The mean age of the participants was 21 years (SD = 3.21), with ages ranging from 18 to 35 years.

The initial sample consisted of 159 students. However, data quality assurance measures resulted in the exclusion of 11 participants. Three were excluded due to responses deviating more than four standard deviations from the mean, indicating potential outliers. Additionally, eight participants were excluded due to response invariability, thereby enhancing the overall reliability of the data.

### 2.2 Instruments

The instruments for this study were adapted from previous tools, specifically to meet two important requirements. First, they needed to be contextualized for a course or subject spanning an entire semester. Second, they had to include a small number of items to ensure ease of application and, consequently, the feasibility of the study.

Self-reported motivation throughout the semester in a given course was assessed using a scale from 1 to 10, where 1 represented low self-reported motivation and 10 represented high self-reported motivation (“Indicate how motivating the course was on a scale of 1 to 10, where 1 is the least motivating and 10 is the most motivating”). This item on motivation allowed for the assessment of the magnitude of motivation in each of the two courses previously identified as the most and least motivating. Although single-item measurements have been criticized in the past, they offer advantages when a general and brief assessment is required (Allen et al., 2022). It is important to note that this measurement does not aim to capture a hypothetical construct but rather to provide a general estimate of students’ self-reported motivation, based on their own understanding of this construct. Since it is a single-item measure, it is not possible to obtain psychometric indicators of validity and reliability.

Affect was measured using a modified version of the Chilean validation of the Positive and Negative Affect Schedule (PANAS) (Dufey and Fernández, 2012). Two psychologists, one specializing in affectivity, and a professor with expertise in teaching, provided judgments for content validity. This adaptation comprised 5 items measuring positive affect (happiness, interest, enthusiasm, confidence, and satisfaction) and 5 items measuring negative affect (embarrassment, fear, sadness, frustration, and anger). Item selection was guided by the relevance of the affect or emotion within the context of various situations encountered in a course. Participants were prompted to indicate the intensity of the affects experienced on average throughout the semester within the course. Responses were scaled from none (0 points) to very much (4 points).

To assess their internal structure, an Exploratory Factor Analysis (EFA) was conducted using a polychoric matrix in the Mplus 8.11 software (Muthen and Muthen, 2024). The procedure followed the guidelines outlined by Lloret-Segura et al. (2014). The WLSMV estimator was employed with GEOMIN rotation. The resulting solution, which demonstrated both theoretical coherence and satisfactory fit indices, consisted of two factors. The RMSEA was 0.063 (CI 90% 0.042–0.085), CFI was 0.994, TLI was 0.989, and SRMR was 0.023. These factors grouped all positive affect items into one factor and negative affect items into another. The negative affect factor exhibited internal consistencies of 0.850 and 0.852 using Cronbach’s alpha and McDonald’s omega, respectively. Meanwhile, the positive affect factor showed internal consistencies of 0.903 and 0.904 using Cronbach’s alpha and McDonald’s omega, respectively.

BPN satisfaction within the context of a course was measured using an *ad hoc* scale specifically developed for the university course setting. Initially, the scale comprised a total of 10 items: three items assessing the Competence need (e.g., “The course challenged me to develop my abilities”), three items evaluating Autonomy (e.g., “Throughout the course, I had the opportunity to freely choose certain topics I wanted to explore further”), and four items addressing Relatedness (e.g., “During the course, I felt valued by the instructor”). Content validity was established through the evaluation of two psychologists specializing in self-determination theory and one psychologist experienced in measurement techniques.

To assess its internal structure, an Exploratory Factor Analysis (EFA) was conducted, following the methodology employed for the previous instrument. This analysis was thus performed using a polychoric matrix with the Mplus 8.11 software, adhering to the guidelines outlined by Lloret-Segura et al. (2014). The WLSMV estimator with GEOMIN rotation was utilized. The best solution consisted of a single factor with 8 items covering the three BPN of the model. The fit indices for this model were as follows: RMSEA of 0.068 (CI 0.021–0.106), CFI of 0.984, TLI of 0.978, and SRMR of 0.060. This factor exhibited an internal consistency of 0.906 for Cronbach’s alpha and 0.910 for McDonald’s omega.

Finally, to assess academic performance, participants were asked to recall and report the average grade obtained in each of the referenced courses: the one they perceived as the least motivating and the one they perceived as the most motivating.

### 2.3 Procedure

All instruments were administered online using Google Forms. The contacted students were invited to participate and were given a flyer that included a QR code to access the form. Before proceeding with the survey, participants were required to review and agree to an informed consent form detailing the objectives of the research, anticipated outcomes, and any possible minimal risks associated with their involvement. This document also identified the principal investigator along with their contact information, and informed participants of their right to withdraw from the study. No personal information, including names or other identifiers, was requested. The research project was previously evaluated and approved by the Scientific Ethics Committee of Universidad de La Serena, in compliance with the ethical principles outlined in the Declaration of Helsinki.

The survey included a sociodemographic questionnaire, followed by several key scales: a positive and negative affect scale, a BPN scale, a question regarding the motivation level, and the average grade obtained during the course. Participants were required to complete these scales twice: once thinking about the most motivating course and once thinking about the least motivating course of the last semester.

It is important to clarify that in Chile most academic programs have rigid curricula with a single option for courses and instructors for all students within a given program. This means that students do not have the option to choose their courses or instructors, except in some cases where elective courses are offered. Elective courses were not considered in this study.

### 2.4 Data analysis

Eleven participants were identified whose responses were either above four standard deviations or exhibited a lack of variability across different items. Consequently, all responses from these participants were excluded. No further data adjustments were made to the matrix. After cleaning the data, descriptive and comparative analyses were conducted using Jamovi 2.3.28 (The Jamovi Project, 2023). Specifically, paired-sample t-tests were performed to compare the levels of the study variables between the courses that participants identified as the most and the least motivating.

To test the hypothesized model, responses from the most motivating and the least motivating courses were combined into a single data set, resulting in a total of 296 data points. This new data set was then subjected to a correlation analysis using Jamovi 2.3.28. Following this, a Structural Equation Modeling (SEM) analysis was conducted using Mplus 8.11, employing the WLSMV estimator.

To evaluate the fit of the hypothesized model, the following indices were used: Chi-square (χ^2^), which assesses the discrepancy between the observed covariance matrix and the one estimated by the model. An associated *p*-value greater than 0.05 indicates good fit; however, this statistic is sensitive to sample size. The Comparative Fit Index (CFI) compares the proposed model with a null model assuming independence among variables. CFI values above 0.95 suggest good model fit. The Tucker-Lewis Index (TLI), also known as the Non-Normed Fit Index, compares the fit of the specified model to that of a null model while penalizing for model complexity. TLI values above 0.95 indicate good fit. The Root Mean Square Error of Approximation (RMSEA) measures the discrepancy per degree of freedom in the model. Following Steiger (2007) recommendation, values below 0.07 indicate a good model fit. The Weighted Root Mean Square Residual (WRMR) evaluates the weighted discrepancy between the observed and estimated covariance matrices. A WRMR value below 1.0 indicates acceptable model fit (DiStefano et al., 2017).

## 3 Results

### 3.1 Description of study variables

Table 1 depicts the means and variability of the studied variables. Overall, as hypothesized, means tend to be higher in the context of the most motivating courses, except for negative affect, which exhibits higher values in the least motivating contexts. The Shapiro–Wilk test reveals that the distribution of the majority of variables deviates from normality.

**Table 1:** General description of the study variables.

Variables	Mean	SD	Min	Max	Shapiro–Wilk	
					*W*	*p*
M –	3.56	1.69	1.00	8.00	0.94	<0.001
M +	8.65	1.31	5.00	10.00	0.86	<0.001
PA –	7.91	4.14	0.00	17.00	0.98	0.033
PA +	15.71	3.02	5.00	20.00	0.95	< 0.001
NA –	9.30	5.09	0.00	20.00	0.97	0.006
NA +	4.78	4.42	0.00	19.00	0.88	<0.001
BPNS –	13.37	6.11	0.00	29.00	0.98	0.084
BPNS +	24.64	5.57	3.00	32.00	0.94	< 0.001
AP –	5.28	0.99	2.50	7.00	0.97	0.005
AP +	6.01	0.78	4.00	7.00	0.93	<0.001

M, self-reported motivation; PA, positive affect; NA, negative affect; BPNS, basic psychological need satisfaction; AP, academic performance. The ‘+’ and ‘–’ symbols indicate whether the variable pertains to the most motivating or the least motivating course, respectively.

### 3.2 Comparisons between the most motivating courses and the least motivating courses

In order to further examine the comparisons of self-reported motivation, positive affect, negative affect, BPN satisfaction, and academic performance in the most motivating and least motivating courses, a paired samples t-test was employed. The analysis revealed statistically significant differences across all variables, accompanied by a large effect size. As observed, the mean values were consistently higher in the most motivating course context for all variables, except for negative affect, which exhibited lower levels (refer to Table 2 for details).

**Table 2:** Study variables comparison in the context of the most and the least motivating course.

Variables	Course				*t*	df	*p*	*d*	95% CI	
	–		+						Min	Max
	*M*	SD	*M*	SD						
M	3.56	1.69	8.65	1.31	−32.5	147	<0.001	−2.67	−3.01	−2.32
PA	7.91	4.14	15.71	3.02	−20.03	147	<0.001	−1.65	−1.89	−1.40
NA	9.30	5.09	4.78	4.42	10.18	147	<0.001	0.84	0.65	1.02
BPNS	13.37	6.11	24.64	5.57	−18.93	147	<0.001	−1.56	−1.80	−1.32
AP	5.27	0.99	6.00	0.78	−8.72	142	<0.001	−0.73	−0.912	−0.54

M, self-reported motivation; PA, positive affect; NA, negative affect; BPNS, basic psychological needs satisfaction; AP, academic performance. The ‘+’ and ‘–’ symbols refer to the most motivating or the least motivating course, respectively.

### 3.3 Correlations between study variables

Table 3 presents the Spearman correlations between the study variables. Every variable significantly correlated with each other. Specifically, positive affect had a significant, high-strength positive correlation with motivation and BPN satisfaction. Similarly, motivation also showed a high-strength positive correlation with BPN satisfaction. Weaker correlation coefficients were seen between the rest of the variables.

**Table 3:** Spearman correlations between the study variables.

	*M*	PA	NA	BPNS	AG
M	—				
PA	0.81*	—			
NA	−0.45*	−0.34*	—		
BPNS	0.79*	0.75*	−0.38*	—	
AP	0.43*	0.34*	−0.44*	0.32*	—

*p < 0.001. M, self-reported motivation; PA, positive affect; NA, negative affect; BPNS, basic psychological needs satisfaction; AP, academic performance.

### 3.4 Structural model of basic psychological need satisfaction, affect, self-reported motivation, and academic performance

**Figure 2:**
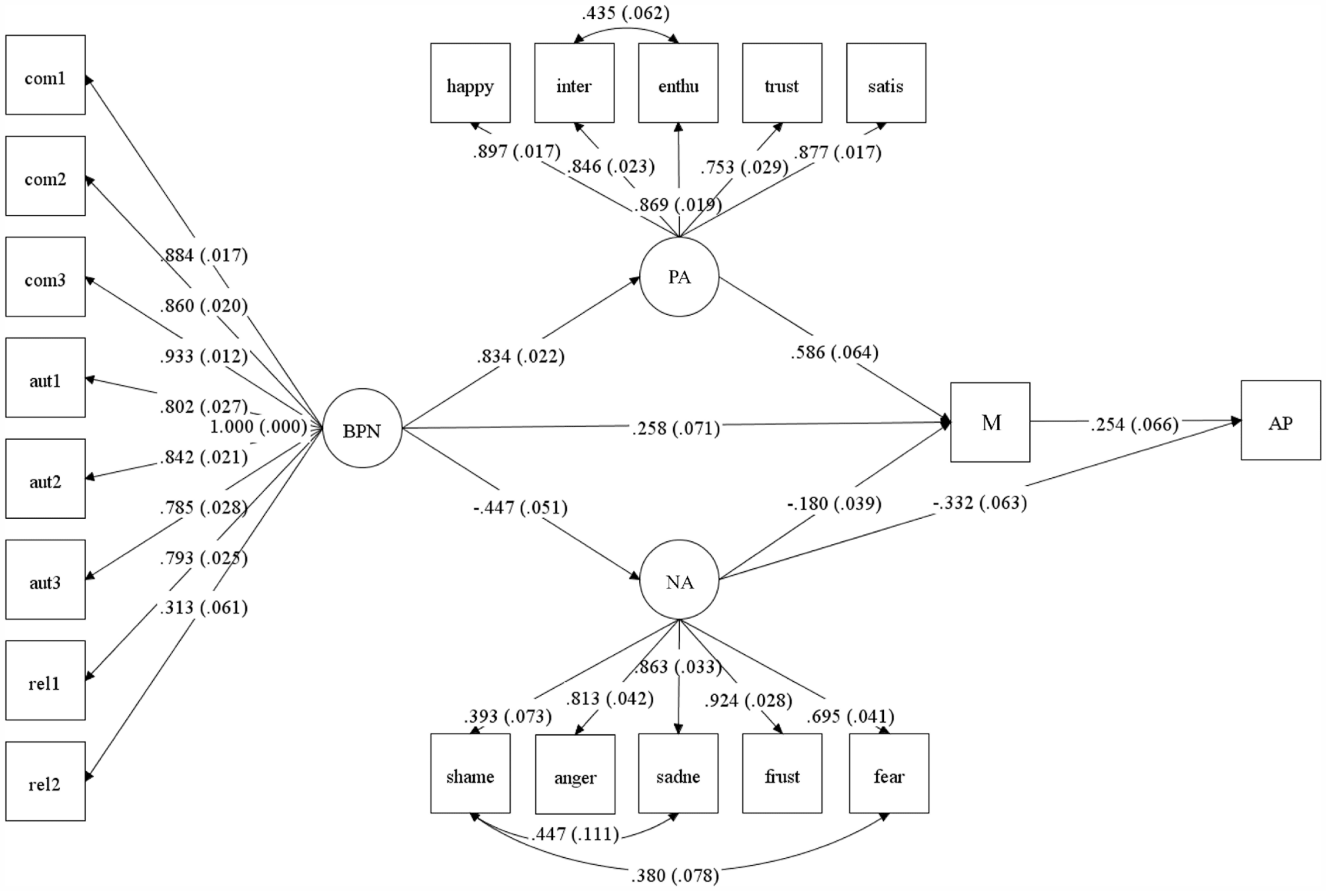
Adjusted general model of association between the study variables. Standardized coefficients are presented outside the parentheses, while standard errors are shown within the parentheses. BPNS, basic psychological need satisfaction; PA, positive affect; NA, negative affect; M, self-reported motivation; AP, academic performance. This Figure was created using Mplus 8.11 (Muthen and Muthen, 2024).

The results of the initial model, shown in Figure 1, indicated a reasonable but not optimal fit to the data (χ2 = 462.183, *p* = 0.0000, CFI =0.976, TLI = 0.973, WRMR = 1.134, RMSEA = 0.079, 90% CI = 0.070 to 0.087). Therefore, it was necessary to evaluate an alternative model (Figure 2). To create a revised structure, new associations between the variables were established. The SEM results indicated that the adjusted model fits the data well, while retaining theoretical consistency with the original model (χ2 = 375.627, *p* = 0.0000, CFI = 0.983, TLI = 0.980, WRMR = 0.977, RMSEA = 0.068, 90% CI = 0.059 to 0.077).

### 3.5 Standardized direct, indirect and total effects

In the adjusted model, all direct effects were significant. BPN satisfaction was positively associated with positive affect (*β* = 0.834, *p* = 0.000, 95% CI = 0.790 to 0.878), and motivation (*β* = 0.258, *p* = 0.000, 95% CI = 0.119 to 0.397), and inversely associated with negative affect (*β* = −0.447, *p* = 0.000, 95% CI = −0.546 to −0.347). Additionally, positive affect was positively associated with motivation (*β* = 0.586, p = 0.000, 95% CI = 0.460 to 0.712), while negative affect was inversely associated with this variable (*β* = −0.180, *p* = 0.000, 95% CI = −0.256 to −0.104). In contrast to the initial model, the adjusted model includes an inverse association between negative affect and academic performance (*β* = −0.332, *p* = 0.000, 95% CI = −0.454 to −0.209). Finally, as expected, motivation was positively associated with academic performance (*β* = 0.254, p = 0.000, 95% CI = 0.124 to 0.384).

Significant indirect effects between the study variables were also examined. As seen in Table 4, BPN satisfaction had a significant indirect effect on both academic performance and motivation, with the latter being the strongest association when positive affect served as a mediating variable (*β* = 0.489, *p* = 0.000, 95% CI = 0.374 to 0.604). Additionally, motivation mediated the relationship between BPN satisfaction and academic performance, both independently (*β* = 0.0.66, *p* = 0.009, 95% CI = 0.017 to 0.115) and in conjunction with positive affect (*β* = 0.124, *p* = 0.001, 95% CI = 0.053 to 0.196) and negative affect (*β* = 0.020, *p* = 0.004, 95% CI = 0.006 to 0.034). Furthermore, academic performance was also indirectly affected by positive affect (*β* = 0.149, *p* = 0.000, 95% CI = 0.065 to 0.233) and negative affect (*β* = −0.046, *p* = 0.002, 95% CI = −0.075 to −0.017) through self-reported motivation, with the latter being the only negative indirect effect found.

**Table 4:** Indirect relations between the study variables.

Indirect effect	β	*p*-value	95% CI
BPNS **→** PA **→** M	0.489	0.000	0.374, 0.604
BPNS **→** NA **→** M	0.080	0.000	0.040, 0.120
BPNS **→** M **→** AP	0.066	0.009	0.017, 0.115
BPNS **→** NA **→** AP	0.148	0.000	0.086, 0.210
BPNS **→** PA **→** M **→** AP	0.124	0.001	0.053, 0.196
BPNS **→** NA **→** M **→** AP	0.020	0.004	0.006, 0.034
PA **→** M **→** AP	0.149	0.000	0.065, 0.233
NA **→** M **→** AP	−0.046	0.002	−0.075, −0.017

BPNS, basic psychological need satisfaction; PA, positive affect; NA, negative affect; M, self-reported motivation; AP, academic performance.

Finally, when considering both direct and indirect pathways, BPN satisfaction had a significant total effect on academic performance (*β* = 0.358, *p* = 0.000, 95% CI = 0.269 to 0.448).

## 4 Discussion

The first objective of the study was to assess differences in BPN satisfaction, positive and negative affect, and academic performance between courses perceived as the most and the least motivating by students. As anticipated, the most motivating courses were associated with greater positive affect, reduced negative affect, higher BPN satisfaction, and better academic performance. These findings are consistent with the principles of SDT, highlighting not only the importance of self-reported motivation and academic performance, commonly associated with students’ academic experience, but also the relevance of BPN satisfaction and affect as equally fundamental factors. Furthermore, a considerable effect size was observed in all comparisons made, suggesting a high probability of replicability of these results in future samples.

Overall, these findings align with previous research emphasizing the fundamental role of BPN satisfaction in fostering motivation and academic performance (Ryan and Deci, 2001; Shang et al., 2024). The significant differences observed in BPN satisfaction, affect, and performance between the most and least motivating courses are consistent with predictions derived from Self-Determination Theory (Ryan and Deci, 2017).

To further explore the relationships between the variables studied, the second objective of this research was to examine the relationship between BPN satisfaction, academic performance, positive and negative affect, and self-reported motivation. While the initial model was theoretically robust, it exhibited a reasonable but suboptimal fit to the data, prompting necessary adjustments. In response, the revised model introduced additional associations, including a notable adjustment: the incorporation of a direct inverse relationship between negative affect and academic performance, which had not been hypothesized in the initial model. As anticipated, the findings revealed that BPN satisfaction predicts academic performance both directly and indirectly, with affect and motivation acting as mediators. These results underscore the importance of designing university courses that satisfy students’ BPN, as they are associated with greater self-reported motivation and improved academic performance—a core principle of SDT.

The results of this study also show that BPN satisfaction has a proportional effect on positive affect, while the opposite occurs with negative affect. Likewise, it was found that positive affect positively predicts self-reported motivation, while negative affect has the opposite effect on it. Analyzing this relationship is quite complex due to the diversity of functions and levels that affect has (González-Arias, 2023). Among these functions, there is the informative role of the body to the brain regarding homeostatic self-regulation at a behavioral level (Roth and Benita, 2023) and the motivational role of affects, which provide energy in response to environmental events (Pekrun et al., 2002). This allows to suggest that an environment that favors BPN satisfaction will involve the experimentation of positive affects, which promote approach behaviors with the purpose of taking advantage of the nourishing opportunities that this environment offers.

Similarly, the results show that students’ self-reported motivation has a direct and positive effect on academic performance. At a theoretical level, the relationship found between these variables can be explained by the facilitating role that motivation plays in the execution of academic activities. The effect of self-reported motivation on academic performance found in this study is consistent with that reported in previous studies (see Liu et al., 2024; Gumasing and Castro, 2023; Méndez-Aguado et al., 2020). However, the scientific literature presents contradictory evidence on this relationship, as some studies have not found a correlation between these two variables (e.g., Muñoz and Correa, 2023), while others have shown that motivation only has an indirect effect on academic performance (see Wu et al., 2020; Kusurkar et al., 2013). The lack of consensus on this issue may be due to limitations inherent in measuring academic performance through grades, which may be affected by reliability problems, variability in assessment criteria, and validity issues (Cain et al., 2022).

As it was previously stated, Achievement Goal Theory (Elliot and Hulleman, 2017) explains that different types of goals lead to different academic outcomes. Given the complex and interconnected systems within which classrooms operate—encompassing schools, homes, communities, and broader societal influences—students’ motives and their perceptions of performance may vary widely (Urdan and Kaplan, 2020). These variations are often shaped by cultural and pedagogical practices, which influence how students prioritize mastery or performance goals. In the Chilean educational context, for instance, it has been found that students in the areas of health and education present a tendency to study because of altruistic reasons, focusing in being capable to treat users effectively (Miranda-Ossandón et al., 2023), which can be linked to a better motivational pattern and thus better academic outcomes. This is relevant for explaining the results of the present study, as most of the sample was composed by students of psychology and pedagogies.

On the other hand, a non-expected inverse association between negative affect and academic performance was found. This suggests that negative affect not only indirectly predicts academic performance through motivation but also has a direct predictive effect. At a theoretical level, this result can be explained considering that negative affect has detrimental effects on cognitive processing, leading to greater attentional and psychological resources consumption and worse cognitive performance (Yang et al., 2023), which is key for achieving satisfactory academic outcomes.

There is no clear consensus on the impact of negative emotions on academic performance. For example, anxiety has been associated with both improved and diminished academic outcomes in students (Mirawdali et al., 2018; Al-Qaisy, 2011). However, a review by Alshareef et al. (2024) found that most good-quality studies indicate anxiety is linked to reduced academic performance. This aligns with the findings of the present study, as negative affects, such as anxiety, may impair memory function, interfere with judgment and cognitive processing, and reduce concentration (Alshareef et al., 2024). Consequently, professors face the critical challenge of reducing students’ experiences of shame, guilt, anger, and other negative affects. Addressing this is essential not only for fostering a healthy academic environment but also for supporting optimal academic performance, as negative affect can significantly disrupt the learning process.

Finally, the results of the present study also indicate that BPN satisfaction indirectly and positively predicts academic performance, aligning with the principles of Self-Determination Theory (SDT). However, some literature suggests a negative relationship between BPN satisfaction and academic performance among university students, arguing that satisfying these needs could reduce the interest in improving grades (Nishimura and Joshi, 2021). In addition, another study indicates that poor academic performance can reduce competence and relationship satisfaction, while grades could limit autonomy by leading students to choose courses that guarantee better grades instead of those more relevant to their learning or personal growth (Chamberlin et al., 2018). These antecedents show the inherent complexity of the relationship between BPN satisfaction and academic performance, which highlights the need for further research in this area.

Overall, the results of this study supports BPN satisfaction as a vitally important factor for the learning process of university students, as it fosters positive affects and decreases negative affects while significantly predicting self-reported motivation, which, in turn, translates into improved academic performance. These findings are consistent with the postulates of SDT, which states that when the satisfaction of students’ BPN is facilitated by promoting their feeling of autonomy, competence, connection and value, positive affects are experienced. Additionally, the inclusion of a direct inverse relationship between negative affect and academic performance emphasizes the importance of exploring not only beneficial determinants in a higher education context but also affective factors that may hinder learning when investigating this theoretical framework.

From a practical perspective, these results stress the importance of educational strategies aimed at reducing negative affect in learning environments. Additionally, they underscore the need to design university courses that foster enriching environments capable of promoting the satisfaction of students’ BPN. This idea is supported by numerous studies demonstrating that the effective implementation of active learning strategies positively impacts BPN satisfaction (Mentzer et al., 2023) and enhances academic performance in university settings (Freeman et al., 2014; Lo and Hew, 2019; Kozanitis and Nenciovici, 2023). Likewise, the way in which the teacher provides feedback on students’ performance is also relevant (Deci and Ryan, 1985). It has been found that feedback through descriptive comments on performance, rather than just grades, generates a higher level of perceived competence, motivation, and academic performance in students (Koenka et al., 2019). Similarly, it has been found that promoting a learning climate that encourages student autonomy is associated with greater satisfaction with their BPN (Levesque-Bristol et al., 2020; Zhou et al., 2023) and motivation (Orsini et al., 2018). Implementing these changes has the potential to improve not only academic outcomes but also the overall well-being of students (Ryan and Deci, 2017; Hayat et al., 2020).

In the educational field, there are approaches that emphasize the theoretical selection and transmission of content and skills, which can lead to a lessened attention to the affective and motivational aspects of students, despite their importance in the learning processes. Therefore, the findings of this study highlight the need for higher education institutions to integrate the promotion of BPN satisfaction into the design of their courses, since this will contribute to achieving their objectives of training well-rounded professionals. Additionally, these results provide empirical evidence that supports SDT in the Latin American university context, which helps to identify the most universal aspects of the theory and those that vary according to the cultural context.

## 5 Limitations and projections

This study has several limitations that must be acknowledged. First, its cross-sectional design restricts the ability to infer causal relationships between the variables. Additionally, the study relied on a small, non-randomized sample composed exclusively of students from a specific region in Chile, potentially limiting the generalizability of the findings to other cultural and educational contexts. Furthermore, the use of self-reported measures introduces the possibility of social desirability bias, which may influence the accuracy of the data. Moreover, data collection was conducted through Google Forms, a method that might have induced selection bias by favoring the inclusion of participants with greater access to digital resources. Finally, the absence of incentives for participants could have affected both the response rate and the quality of the data.

Future studies could adopt longitudinal designs to examine how interactions among BPN satisfaction, affect, and motivation evolve over time. Expanding the sample to include diverse regions and cultural contexts would also be valuable to explore cross-cultural variations. Moreover, future research could employ mixed methods, combining qualitative and quantitative approaches to capture a more comprehensive understanding of student experiences and contextual dynamics in higher education.

The use of a general BPN satisfaction instrument, rather than an instrument that assesses competence, relatedness, and autonomy individually, limited the conclusions obtained in the analysis. It is suggested that future studies use instruments that separately assess BPN satisfaction to determine whether the course context contributes equally to the satisfaction of all BPN satisfaction dimensions or whether certain factors affect these dimensions in a differentiated way. Finally, it would be advisable to conduct studies that more precisely discriminate between different types of motivation, using instruments tailored to the course context.

## 6 Conclusion

The findings of this study reveal that in courses perceived as most motivating, students experience higher positive affect, lower negative affect, higher BPN satisfaction, and better academic performance. Furthermore, BPN satisfaction was found to both directly and indirectly predict self-reported motivation, mediated by both types of affects, while negative affect and motivation directly predict academic performance. These results contribute to a better understanding of how BPN satisfaction influences the academic performance of university students. They also reinforce the usefulness of SDT in explaining motivational and affective phenomena in higher education, underlining that BPN satisfaction not only improves the academic experience, but also highlights the importance of educational environments incorporating these principles.

## Data Availability

The raw data supporting the conclusions of this article will be made available by the authors, without undue reservation.
